# Whole Genome Amplification of Day 3 or Day 5 Human Embryos Biopsies Provides a Suitable DNA Template for PCR-Based Techniques for Genotyping, a Complement of Preimplantation Genetic Testing

**DOI:** 10.1155/2017/1209158

**Published:** 2017-06-22

**Authors:** Elizabeth Schaeffer, Bruno López-Bayghen, Adina Neumann, Leonardo M. Porchia, Rafael Camacho, Efraín Garrido, Rocío Gómez, Felipe Camargo, Esther López-Bayghen

**Affiliations:** ^1^Laboratorio de Investigación y Diagnóstico Molecular, Instituto de Infertilidad y Genética, Ingenes, Ciudad de México, Mexico; ^2^Departamento de Genética y Biología Molecular, Cinvestav-IPN, Ciudad de México, Mexico; ^3^Departamento de Toxicología, Cinvestav-IPN, Ciudad de México, Mexico

## Abstract

Our objective was to determine if whole genome amplification (WGA) provides suitable DNA for qPCR-based genotyping for human embryos. Single blastomeres (Day 3) or trophoblastic cells (Day 5) were isolated from 342 embryos for WGA. Comparative Genomic Hybridization determined embryo sex as well as Trisomy 18 or Trisomy 21. To determine the embryo's sex, qPCR melting curve analysis for SRY and DYS14 was used. Logistic regression indicated a 4.4%, 57.1%, or 98.8% probability of a male embryo when neither gene, SRY only, or both genes were detected, respectively (accuracy = 94.1%, kappa = 0.882, and *p* < 0.001). Fluorescent Capillary Electrophoresis for the amelogenin genes (AMEL) was also used to determine sex. AMELY peak's height was higher and this peak's presence was highly predictive of male embryos (AUC = 0.93, accuracy = 81.7%, kappa = 0.974, and *p* < 0.001). Trisomy 18 and Trisomy 21 were determined using the threshold cycle difference for RPL17 and TTC3, respectively, which were significantly lower in the corresponding embryos. The Ct difference for TTC3 specifically determined Trisomy 21 (AUC = 0.89) and RPL17 for Trisomy 18 (AUC = 0.94). Here, WGA provides adequate DNA for PCR-based techniques for preimplantation genotyping.

## 1. Introduction

Use of assisted reproduction technology, such as in vitro fertilization (IVF), has become more widespread over the past decade. One of the main causes is due to women waiting later in life to get pregnant; however, IVF alone cannot compensate for the lower fertilization rates that are associated with advanced age [1]. During assisted reproductive treatments, it is highly suggested for patients to complement IVF with Preimplantation Genetic Testing (PGT) to assess embryos for possible aneuploidies, genetic defects, or diseases.

A variety of methods are currently available for PGT, with each having their advantages and disadvantages. For example, Comparative Genomic Hybridization (CGH) and Fluorescent In Situ Hybridization (FISH) require days rather than hours to perform, meaning embryos have to be frozen. Moreover, these techniques come with high procedural costs [2]. The most important concerns for PGT are the reliability of prediction, method error rates, ex vivo embryo maintenance, and, to a lesser extent, procedural costs. As for reliability of prediction and method error rates, allele drop-out (ADO) has been shown to diminish or remove the signal for multiple techniques [3] leading to incorrect genotype assignment. On the other hand, false allele (FA), an amplification artifact that causes the appearance of new allele, is also leading to erroneous identification of the chromosome make-up. Polymerase Chain Reaction (PCR) is a rapid, relatively inexpensive, and highly sensitive technique, making it a suitable option for specific genotyping. Interestingly, the initial step of CGH for PGT requires the whole genome to be amplified, which allows further analysis of many PGT targets. Despite this fact, to our knowledge, information whole genome amplification (WGA) coupled with PCR-based strategies is a technology still being optimized which needs further assessment ([3–7]).

Some of the most characterized PCR-based techniques focus on determining the sex of tissues for forensic science or for embryos post implantation. Currently, most methods for sex determination use the detection of genes that are only associated with the Y-chromosome, such as sex determining region Y (SRY) and DYS14, a marker found in the intron of the TSPY gene [8, 9]. SRY is a single copy gene [10], whereas TSPY is a multicopy gene [11, 12]; therefore, differences in the detection capabilities for each gene could be expected. Moreover, the initial amount of genetic material could significantly affect the detection of SRY, especially when starting with a single cell. Another well-characterized system is examining the amelogenin genes. The amelogenin genes, which are present on both the X-chromosome (AMELX) and the Y-chromosome (AMELY), have been used to determine sex in cattle [13], sheep, and deer [14] as well as in other species of the Bovidae family [14]. In humans, both genes are nearly identical; however, there is a 6 bp insert in intron 1 of AMELY. Using PCR, Shadrach et al. demonstrated that amplifying this region with a single PCR reaction produced a 104 and 110 bp amplicon for AMELX and AMELY, respectively [15]. Thus, the presence of two amplicons suggests male tissue, whereas one amplicon suggests female tissue.

Two common genetic abnormalities with high prevalence during IVF procedures are Trisomy 21 (Down syndrome) and Trisomy 18 (Edwards syndrome). For Trisomy 21, current methods include examining the Down Syndrome Critical Region, located on chromosome 21, which contains many genes whose duplication lead to the phenotypic features of Down syndrome, such as Tetratricopeptide Repeat Domain 3 (TTC3) gene [16, 17]. Recently, quantitative amplification of the TTC3 gene was shown to discriminate between subjects with Down syndrome from normal subjects; however, this study focused on prenatal and neonatal sources [16]. For Trisomy 18, investigators can examine the ribosomal protein gene (RPL17), located at chromosome 18q21.1-q21.1 [18]; however, the association between RPL17 and Trisomy 18 detection has yet to be investigated.

For humans, PCR-based techniques for AMEL, SRY, and DYS14 have been implemented to determine the embryo's sex after implantation; however, these methods require a significant amount of genomic DNA. The possibility of examining these genes before embryo implantation has yet to be fully investigated; therefore, we tested the ability for the detection of these amplicons when using the WGA product as the template. Here, we examined SRY, DYS14, AMELX, AMELY, TTC3, and RPL17 using WGAs from a single blastomere (Day 3) or trophoblastic (Day 5) biopsies, to determine the two important trisomies and the sex of the embryos from patients undergoing IVF treatments.

## 2. Materials and Methods

### 2.1. Study Patients and Ethical Approval

Eighty-three women (age range: 21–47 years), undergoing IVF in Mexico City, were included in this cross-sectional study. Patients were clinically evaluated according to a standardized protocol that included personal and family clinical history. The protocol was approved by the Ethics Committee of the Ingenes Institute. Written informed consent was obtained from all patients, conducted in accordance with the Declaration of Helsinki.

### 2.2. In Vitro Fertilization and Pregnancy

All patients were subjected to controlled ovarian stimulation for 10 days with gonadotrophin-releasing hormone agonists and antagonists. Ovarian response was assessed measuring serum estradiol levels and follicular development was evaluated by ultrasound examination. Oocyte retrieval was conducted 20 hours after human chorionic gonadotropin administration with ultrasound guidance. The follicles aspirated ranged between 2 and 43 (average = 12.7 ± 7.7) and the embryos obtained ranged between 1 and 10 (average = 3.7 ± 2.3) per patient. The average fertilization rate was 72.3 ± 20.4%. An embryologist monitored and recorded all information about fertilization rates, embryo development, and embryo morphology for each oocyte.

### 2.3. Embryo Biopsy (Day 3 and Day 5) and Collection of Control Samples

For Day 3 embryos, we utilized the S-biopsy method. The S-biopsy method is a simplified displacement method in combination with laser-assisted hatching for the removal of a blastomere and is performed on cleavage stage embryos [19]. Briefly, a laser (Octax, PL, Europe) was used to create a thin funnel in the zona pellucida adjacent to the desired blastomere. Next, the blastomere was extracted by aspirating the complete embryo with a 140 *μ*m stripper capillary micropipette, leading to the ejection of the blastomere. The blastomere was then placed into a 0.2 *μ*L PCR tube and separated for further analysis.

For Day 5 embryos (expanded blastocyst stage containing 50 to 150 cells), a laser (Octax, PL, Europe) was used to create a thin funnel in the zona pellucida on the opposite side to the inner cell mass. Blastocysts were incubated for a further 2-3 hours to allow blastocoele expansion and herniation of the trophectoderm cells from the zona, at which time the embryo was placed into 20 *μ*L of medium (Vitrolife) under oil for biopsy. Applying gentle suction with the biopsy pipette (MBB-FP-SM-35, Origio, Malov, Denmark), the trophectoderm cells were encouraged to herniate from the zona. Four to five trophectoderm cells were dissected from each of the blastocysts using four laser pulses of 3-minute duration. 10–15 cells were retrieved, washed, and placed into a 0.2 *μ*L PCR tube.

For the control samples, 37 adults (12 females and 25 males) were randomly selected and buccal samples were collected. As for the control samples for Trisomy detection, buccal samples were collected from 11 normal adults (5 females and 6 males) and 4 with Down syndrome (1 female and 3 males). Using dilution and micromanipulation, 10–15 cells were isolated and placed into a 0.2 *μ*L PCR tube. These samples underwent the same WGA process as Day 3 and Day 5 embryo biopsies. Furthermore, blood samples were collected to provide a sufficient amount of genomic DNA, not requiring a WGA step for analysis, using the Wizard Genomic DNA Purification Kit (Promega) according to manufacturer's instructions.

### 2.4. WGA and CGH

Each sample (Day 3, Day 5, or control samples) was amplified using the SurePlex amplification system (BlueGnome, San Diego, CA, USA) according to the manufacturer's instructions. CGH was carried out using the 24Sure V3 microarray (Illumina, San Diego, CA, USA) using the protocol described by Fragouli et al. [20, 21]. The amplified DNA was fluorescently labelled (Fluorescence Labelling System, BlueGnome). The samples were coprecipitated, denatured, and analyzed by array hybridization. The hybridization time was 16 hours. A laser scanner (InnoScan 710, Innopsys, Carbonne, France) was used to excite the fluorophores and read the hybridization images. The hybridization images were stored in TIFF format and analyzed by the BlueFuse Multi-Analysis software (BlueGnome) using criteria and algorithms recommended by the manufacturer. With this approach, it was possible to determine the chromosome constitution of each embryo. Additionally, X- and Y-chromosome hybridization allowed the classification of the embryos as either male (hybridization of Y-chromosome probes) or female (no hybridization of Y-chromosome probes, coupled with higher hybridization of X-chromosome probes). CGH data are available in the public repository Gene Expression Omnibus (GEO accession number GSE97386 for Y-chromosome and GSE97903 for Trisomy at https://www.ncbi.nlm.nih.gov/geo/query/acc.cgi?acc=GSE97386).

### 2.5. Genotyping via qPCR

**Figure 1:**
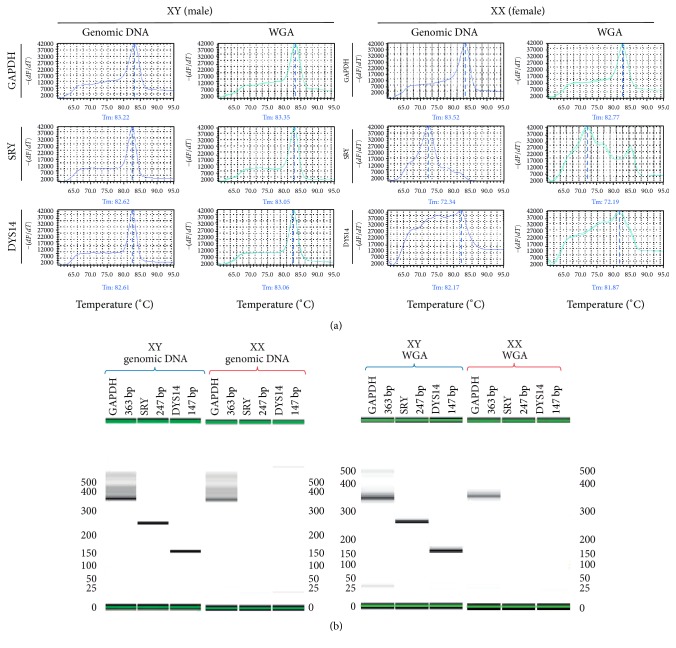
GAPDH, SRY, and DYS14 melting curve analysis. Isolated genomic DNA from control adults (Genomic) and WGA from a blastomere were used as templates for GAPDH, SRY, and DYS14 qPCR reactions. Representative data for males and females are shown. (a) Melting temperature peaks were determined by negative first derivatives (−*dF/dT*) plot. These plots were used for identification of the embryo's sex. Melt curve data was extracted from the StepOne software and used to create graphs. Representative graphs for each gene and sample type are shown. (b) PCR products were resolved through capillary electrophoresis using the BioAnalyzer Labchip GX. Products showed a single band corresponding to the predicted base pair length.

Primers for GAPDH, SRY, DYS14, TTC3, RPL17, and hydroxy-delta-5-steroid dehydrogenase and 3 beta- and steroid delta-isomerase 2C (HSDB) are presented in Table 1 [22–26]. Primers were first validated by amplifying genomic DNA from adult samples. The regions of interest were amplified using the KAPA SYBR® FAST qPCR system. Each 10.0 *μ*L reaction consisted of 5.0 *μ*L of Universal PCR Master Mix, 4 pM of each primer, and 200 to 250 ng of purified WGA. The mixture was vortexed for 5 seconds and then centrifuged for 5 seconds. The PCR conditions were 10 minutes at 98°C, followed by 40 cycles of 30 seconds at 95°C, 30 seconds at 60°C, and 1 minute at 72°C, followed by 10 minutes at 72°C using the StepOne Plus instrument (Thermofisher). All the PCR products were resolved through capillary electrophoresis using the BioAnalyzer Labchip GX (Caliper). The products showed a single band corresponding to the predicted base pair length. Moreover, the bands were cloned and analyzed via sequencing to verify their identity. SYBR Green was used during amplification to construct melting curves that were analyzed to verify if the peaks corresponded with theoretical melting temperatures for each amplicon (Figure 1).

For quantitative genotyping, a subset of 43 embryos biopsied on Day 5 from 31 patients was analyzed for PGT. The embryos were separated into 3 groups based on CGH results (normal, Trisomy 21, or Trisomy 18). For quality control, each sample was run as a standard curve for TTC3, RPL17, and HSDB (endogenous control) with 3.75, 7.5, and 15 ng of purified WGA. Under ideal conditions, the slope of the log WGA concentration to *C*_*T*_ should be −3.3 ± 0.4, to have all three fragments amplified at comparable efficiency. For result analysis, the reaction mixture was the same as above with 15 ng of purified WGA. Each reaction was subjected to 1 cycle of 95°C for 10 seconds, followed by 40 cycles of 95°C for 3 seconds, 58°C for TTC3, and RPL17 or 62°C for HSDB for 30 seconds. Lastly, a melting curve stage (95°C for 15 seconds, 60°C for 1 minute, and 95°C for 15 seconds) was constructed to determine the purity. For result analysis, *C*_*T*_ value considered was that obtained from the reactions containing 15 ng of purified WGA.

### 2.6. Determination of AMELX and AMELY

**Figure 2:**
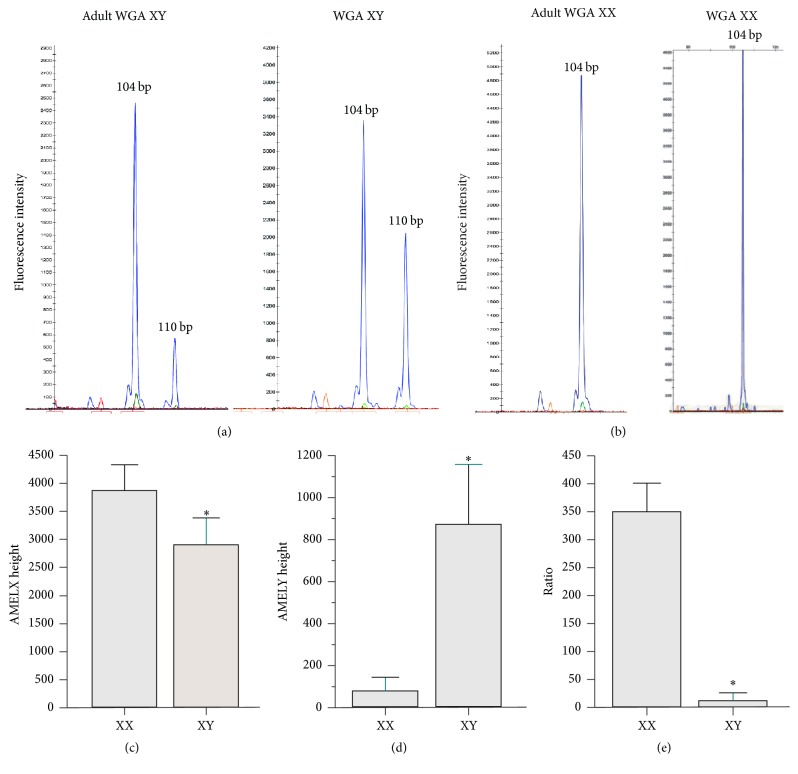
Capillary electrophoresis analysis of amelogenin intron 1. Male and female adult DNA, isolated from 10–15 cells from a buccal sample, were prepared under the same conditions as DNA isolated from Day 5 embryos. PCR products were separated by capillary electrophoresis to confirm AMELX (104 bp) and AMELY (110 bp) for male (a) and female adults (b). Comparison of the AMELX (c), AMELY (d), and peak height and X/Y ratio (e) for Day 3 and Day 5 embryo biopsies. The height of the bar is the average and the error bars represent the 95% CI. *∗* indicates a significant difference between groups (*p* < 0.05).

To determine AMELX and AMELY (primers in Table 1), an initial PCR reaction, containing 5 *μ*L of Universal PCR Master Mix, 4 pM of each primer, and 200–250 ng of purified WGA, was prepared. The PCR conditions were the same as for GAPDH, SRY, and DYS14. A secondary PCR reaction was run with 1 *μ*L of the first amplification product in 0.2 mL PCR tubes containing a similar reaction mix, but now including the forward primer for AMEL conjugated with 6-carboxyfluorescein. Amplicons were analyzed by capillary electrophoresis on the ABI Prism 3130XL Genetic Analyzer using the GeneMapper ID v.3.2 software (Applied Biosystems, Carlsbad, CA, USA). All samples were analyzed with an internal control and an internal size standard (GeneScan-500LIZ, Applied Biosystems, Carlsbad, CA, USA). According to the recommendations of Szibor et al. [27], the genotyping was performed by comparing to the female control DNA 9947A (Promega Corporation, Madison, WI, USA) [28]. The presence of 1 peak at 104 bp was presumed to be indicative of a female genotype (Figure 2(a)), whereas the presence of two peaks at 104 and 110 bp was presumed to be male (Figure 2(b)).

### 2.7. Statistical Analysis

Rates for ADO and FA were calculated according to Broquet and Petit [29]. The Shapiro-Wilk test was used to determine if the data were normally distributed. Either the Mann–Whitney *U* test, *T*-test, or ANOVA with a post hoc Bonferroni test was used to examine differences between groups. *p* values < 0.05 were considered significant. Receiver operating characteristic (ROC) analysis was performed to determine the specificity and sensitivity of AMELX and AMELY genes as an indicator of male embryos and for TTC3 and RPL17 to analyze Trisomy 21 and Trisomy 18. The area under the curve (AUC) was measured to determine the degree of predictability. Logistic regression was used to assess the association between SRY, DYS14, or both genes and the embryo's sex. Cohen's kappa was calculated to determine the level of agreement between the index and the standard (CGH). All analyses were carried out with the Statistical Package for the Social Sciences software (SPSS, v. 22.0, Chicago, IL USA).

## 3. Results

### 3.1. Study Characteristics

Three hundred and forty-two embryos from 83 women were considered for this study. The embryos were randomly distributed for either the GSD index analysis (*n* = 214) or the AMEL analysis (*n* = 128). Six samples showed poor or no amplification at the WGA step and 8 samples showed no amplification of GAPDH, resulting in the loss of 14 embryos for analysis. Using CGH, 146 embryos were determined to be male, whereas 182 embryos were female. For detecting Trisomy 21 or Trisomy 18, a subset of 43 embryos from 31 women was considered for this study. Using CGH, 21 embryos were normal, whereas 9 embryos had a gain in chromosome 18 and 13 embryos had a gain in chromosome 21.

### 3.2. GSD Index Correlated with the Embryo's Sex

As part of the amplification of SRY, DYS14, and GAPDH, melting curves were produced. Melting curves produced using embryonic WGA DNA as template were compared to melting curves produced using adult genomic DNA. All peaks corresponded to the predicted melting temperature (Figure 1(a)). Moreover, when PCR products were separated by capillary electrophoresis, the predicted band size was observed, indicating the accuracy of the method (Figure 1(b)). The presence of GAPDH indicated a successful amplification. If SRY, DYS14, or both genes were present, the embryos were considered male* (GSD index)*. 52.0% of the samples were negative for either SRY or DYS14, suggesting a female embryo (Table 2). On the other hand, 41.2% of the samples were positive for both SRY and DYS14, suggesting a male embryo. Interestingly, 14 samples were positive for only DYS14 and negative for SRY. Logistic regression analysis was performed to evaluate the association between the GSD index and the sex of the embryo. Specifically, SRY and DYS14 together were highly associated with male embryos (Table 2). This resulted in a 4.4% probability of an embryo being male when SRY and DYS14 are not present; however, the probability increased to 57.1% or 98.8% when there was a positive detection for DYS14 or both genes, respectively. When the GSD index was compared to the CGH data, the test was highly accurate (test accuracy = 94.1%, kappa = 0.882, and *p* < 0.001). For SRY, no ADO or FA was seen with data from the adult controls; however, for the embryos, the ADO and FA rates were 13.5% and 0.5%, respectively. For DYS14, no ADO or FA was seen with the control data, whereas, for the embryos, the ADO and FA rates were 5.2% and 3.4%, respectively.

### 3.3. AMELY Gene Is Highly Predictive for Male Embryos

For analysis of the AMEL genes, using the control WGA samples, the male adult samples consistently had two peaks at 104 and 110 bp (Figure 2(a)), whereas the female adult samples only produced one peak at 104 bp (Figure 2(b)). This was in agreement with the genomic samples, suggesting that starting with 10–15 cells did not affect the sensitivity of the method. All of the samples used for the AMEL analysis had suitable genome amplification.

**Figure 3:**
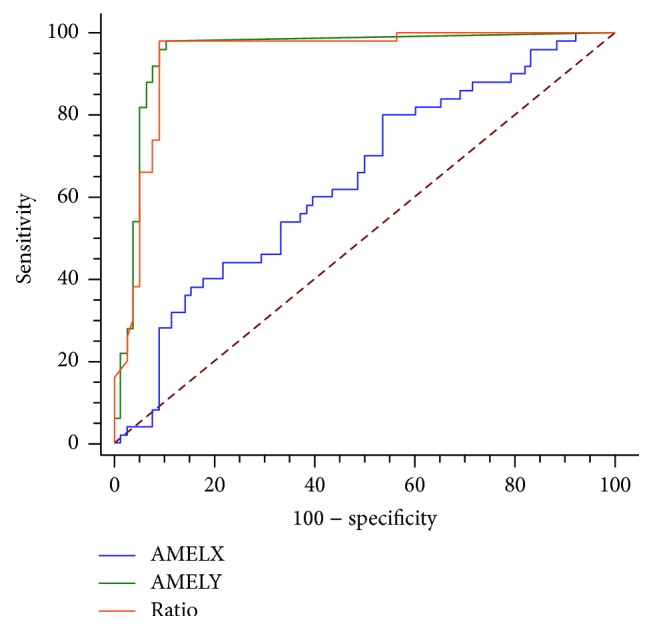
Receiver operating characteristic curve for amelogenin genes in identifying male embryos. AMELX (blue), AMELY (green), and the peak height ratio (orange) were assessed.

CGH determined that 39.1% of the 128 embryos were male. For AMELX, the peak height was statistically higher in females, while the AMELY peak was higher in males (Figures 2(c) and 2(d), resp.). For AMELY, 8 female embryos had a pronounced peak and 1 male embryo failed to produce a peak. Due to this situation, we examined the peak height ratio and determined that the ratio was significantly higher in females (Figure 2(e)). For AMELX, the peak height was a weak indicator of the embryo's sex (AUC = 0.64, 95% CI: 0.54–0.74, and *p* < 0.01; Figure 3). The AMELY peak height and the peak height ratio, on the other hand, were excellent indicators of the embryo's sex (AUC = 0.95, 95% CI: 0.91–0.99 and AUC = 0.94, 95% CI: 0.90–0.98, resp., *p* < 0.01). Using the ROC curve, a cutoff value of 23.5 for the AMELY peak height was determined (Youden index = 0.877, sensitivity = 98%, and specificity = 90%). When the samples were reanalyzed with this cutoff, the test was exceptionally predictive (test accuracy = 93.0%, kappa = 0.856, and *p* < 0.001). A cutoff value of 21.8 was determined for the peak height ratio (Youden index = 0.890, sensitivity = 98%, and specificity = 91%), which also indicated a highly predictive test (test accuracy = 93.8%, kappa = 0.871, and *p* < 0.001). For AMELY, ADO was 2.0% and FA was 6.3% for the embryo data.

### 3.4. Trisomy 18 and Trisomy 21 Detection

For TTC3, a gene present in chromosome 21, the normal group and Trisomy 18 group had similar threshold cycle values; however, Trisomy 21 group had a threshold cycle about 1.4 cycles lower than the normal group (*p* < 0.001, Table 3), suggesting the presence of a gain in chromosome 21. *C*_*T*_ difference for TTC3 specifically determined Trisomy 21 (AUC = 0.89, 95% CI: 0.74–1.00, and *p* < 0.001) and not Trisomy 18 (AUC = 0.66, 95% CI: 0.36–0.96, and *p* = 0.211). *C*_*T*_ cutoff value of 3.85 was calculated for TTC3 to determine Trisomy 21 (Youden index = 0.886, sensitivity = 92.3%, and specificity = 96.3%). Using this cutoff, the test was highly accurate (accuracy = 95.0%, kappa = 0.840, and *p* < 0.001). For RPL17, a gene specific for chromosome 18, Trisomy 18 group had a threshold cycle about 1.7 cycles lower than the normal group (*p* < 0.05). This *C*_*T*_ difference for RPL17 specifically determined Trisomy 18 (AUC = 0.94, 95% CI: 0.86–1.00, and *p* < 0.001) and not Trisomy 21 (AUC = 0.55, 95% CI: 0.35–0.74, and *p* = 0.644). *C*_*T*_ cutoff value of 0.985 was calculated for RPL17 to determine Trisomy 18 (Youden index = 0.801, sensitivity = 88.9%, and specificity = 91.2%), suggesting an exceptional test (accuracy = 90.7%, kappa = 0.742, and *p* < 0.001). A graphic description is presented in Supplementary Figure in Supplementary Material available online at https://doi.org/10.1155/2017/1209158. Overall, these data suggest the possible use of PCR-based techniques and threshold cycle differences to determine genetic abnormalities within hours of an embryo biopsy.

## 4. Discussion

In this work, we attempted to assess the ability to couple PCR-based techniques with WGA to determine its prognostic capability for preimplantation genotyping. We selected as our models the two well-characterized sex determination systems. For IVF treatments, determining the sex of the embryo is not a procedural concern and is normally determined with PGT when assessing sex-linked genetic diseases. However, under certain other circumstances, knowing the sex of the embryo is desired. But with high PGT costs and lengthy procedural times, cheaper and faster alternative techniques are still needed. Here, we demonstrate that two assays, examining the AMEL genes by Fluorescent Capillary Electrophoresis and examining the SRY and DYS14 genes by melting curve analysis are highly accurate at determining the embryo's sex. Moreover, we tested the ability of qPCR to determine Trisomy 18 and Trisomy 21. When using TTC3, we could specifically determine Trisomy 21, and when using RPL17, we could specifically determine Trisomy 18.

The two Y-chromosome regions, SRY and DYS14, were demonstrated to specifically indicate the sex of the embryo when assessed by melting curve analysis. As seen with other studies, using both genes demonstrated a marked improvement of sexing the fetus [12, 30–32]; however, these studies used maternal plasma as the source. To our knowledge, this is one of the first studies to examine the SRY and DYS14 regions to sex an embryo before implantation. Moreover, our results demonstrate similar efficiencies as studies which have used cell-free fetal DNA. Eleven male embryos, all having varying degrees of aneuploidies, were misidentified. They were negative for both markers, suggesting the loss of the Y-chromosome. Four had CGH-confirmed loss of the Y-chromosome. Interestingly, six female embryos were positive for DYS14 and not SRY. This result was also observed with White et al., in which, using qPCR, they detected low levels of DYS14 in female fetuses [33].

The use of the AMEL genes to determine the sex of tissues and fetuses in utero is well documented. However, due to limited genetic material, its preimplantation use remains limited. Here, we used WGA to increase the amount of genetic material and determine that, with this additional step, examining AMEL gene specifically predicted the sex of the embryo. Interestingly, we had one male embryo that was negative for the AMELY gene. This could be due to the presence of an AMELY-negative male. In Mexico, the rate of AMELY-negative males was reported to be 0.08% [34]. However, this is the only report to focus on Latin America and the reported rate is lower than other regions, such as India [35, 36], Singapore [37], and Malaysia [36], which can range between 0.88 and 3.60%.

Six of the female embryos were positive for AMELY. Stapleton et al. reported a family in which three female members had the AMELX and the AMELY genotypes, only possessing a small portion of the Y-chromosome [38]. Another possibility is that these embryos could represent the XX male genotype, in which a portion of the Y-chromosome translocated to the X-chromosome [39, 40]. Moreover, three of the six embryos were identified as having an additional X-chromosome. Monitoring translocation events was outside the scope of this research and was not determined. It is expected to have higher rates of sex chromosome-linked disorders among IVF treatments and women of advanced age [41, 42], which could explain our results. Nevertheless, our results are consistent with the CGH data, indicating that examining AMELX and AMELY to identify an embryo's sex is possible, before implantation.

We also examined the threshold cycle difference associated with two well-characterized trisomies. Interestingly, for either gene the expected cycle difference of 0.5 cycles was not achieved. For TTC3 the difference was 1.4 cycles and for RPL17 was 1.7 cycles. This suggests that the WGA step may not be amplifying the genome equally or evenly. Additionally the difference between normal and trisomic embryos could be due to copy number variation. Goodrich et al. demonstrated that varying levels of mosaicism could affect the detection of Trisomies 13, 15, and 18. Only high levels of mosaicism, ranging from 33% to 83%, allowed for reliable detection of the selected aneuploidies [43]. To address this issue, we plan to include multiple chromosome-specific targets in future studies. Nevertheless, the results show that for Trisomy 18 and Trisomy 21, the threshold cycle was less than the control, indicating an alternative mechanism for a quick and less expensive detection of genetic abnormalities.

Other factors that could lead to discordance between the experimental techniques and the CGH results are ADO or FA. Many technical issues are associated with WGA, from the type of cells used (blastomere versus trophectoderm [7]), to the number of cells used for the WGA step [6], to the quantity and quality of DNA, all of which can impact ADO and FA rates. Other studies have shown that ADO rates for WGA based techniques range between 1.0 and 27.7% and can be as high as 50% [3–7]. Here, minimizing these technical issues, our ADO rate was 2.0% for AMELY (single copy gene), 5.2% for DYS14 (multiple copy genes), and 13.5% for SRY (single copy gene). On the contrary, ADO was not found among the control sample. The difference between the ADO rates for SRY and DYS14 suggests that multiple copy genes could decrease the ADO rate. However, AMELY, which is a single copy gene, had a lower ADO rate. This could be accounted by the greater effort to optimize AMEL detection for forensic sciences, even though the AMELY efficiency remains questionable. It could also be accounted by the different methods to detect SRY and DYS14 (qPCR melting curve analysis) and AMELY (Fluorescent Capillary Electrophoresis). Moreover, for the GSD index, both SRY and DYS14 were required to achieve a 98.8% probability that the embryo was male. For us, the DYS14 only embryos receive no classification in the clinical setting. None of the controls demonstrated FA for AMELY, SRY, or DYS14, whereas the embryos had FA rates of 6.3, 0.5, and 3.4%, respectively. Other studies have shown similar FA rates, ranging between 0.04 and 3.35% [4–6]. Lastly, the variation in the AMELX and AMELY peaks could be more explained by PCR efficiency or failure.

This study has a few limitations. First, we are basing the accuracy of our methods on the CGH microarray, which in itself has a conservative estimated error rate of 2–9% [44, 45]. According to our own data, our system has 3% error rate. It would be better to associate the results of the two assays with late-stage ultrasound or live births. However, due to the regulations in Mexico, in which single embryo transfers and selecting embryos based on their sex is only permitted under certain situations, this is unfeasible. Second, disagreements between the two methods and CGH microarray data could be due to rearrangements or mutations that alter primer efficiency. Third, the assays are solely qualitative and cannot distinguish between any sex chromosome disorders, such as Klinefelter's syndrome, Turner syndrome, Triple X, and XYY. We posit that qPCR could overcome this issue in a similar fashion as seen with detection of Trisomy 18 and Trisomy 21. To be certain of multiple chromosomes, we suggest the use of multiple targets; however, more studies are required.

## 5. Conclusion

Here, we demonstrate that an initial whole genome amplification step provided a sufficient amount of DNA to assess genetic abnormalities of an embryo by different PCR-based techniques: capillary electrophoresis, melting curve analysis, and differential qPCR. These techniques were applied before transfer of an embryo into the uterus during IVF, suggesting a practical use of qPCR with a WGA template for testing. This method provides an alternative to CGH microarray analysis, thus reducing cost and time.

## Supplementary Material

Supplementary Figure 1 illustrates CGH results, as well as melting curve analysis and cycle threshold difference for representative examples of Trisomy 18 and Trisomy 21 embryos.

## Figures and Tables

**Table 1:** qPCR primers.

Gene	Forward primer	Reverse primer	Size	Ref.
GAPDH	5′-TTTAACTCTGGTAAAGTGGATATTG-3′	5′-CTGTTGTCATACTTCTCATGGTT-3′	363 bp	This work
SRY	5′-GCTGGGATACCAGTGGAAAA-3′	5′-TCTTGAGTGTGTGGCTTTCG-3′	247 bp	[22, 23]
DYS14	5′-CATCCAGAGCGTCCCTGG-3′	5′-TTCCCCTTTGTTCCCCAAA-3′	147 bp	[24]
AMEL	5′-CCCTGGGCTCTGTAAAGAATAGTG-3′ (6-carboxyfluorescein labeled)	5′-ATCAGAGCTTAAACTGGGAAGCTG-3′	106 bp (XX) 110 bp (XY)	[25]
TTC3	5′- GAATACTTTGATGATTGCCAACAG-3′	5′-TCACTAGAATACTGCTTCGAGAC-3′	141 bp	This work
RPL17	5′-CCCCACTTAGATGTACATAGCC-3′	5′-TGGAGGACTTCAGCTTATTCTG-3′	236 bp	This work
HSD3B2	5′-CCCACTCCATACCCGTACAG-3′	5′-GTAGAGAACTTTCCAACACTTGAC-3′	206 bp	This work

**Table 2:** Melting curve analysis of SRY and DYS14 to determine embryo sex.

Gene	Female		Male		*p*	Kappa	OR	95% CI
	Correct	Incorrect	Correct	Incorrect				
SRY	107	1	83	13	<0.001	0.861	683	88–5328
SRY + DYS14	101	7	91	5	<0.001	0.882	262	81–856

**Table 3:** Detection for Trisomy 18 and Trisomy 21 for embryo samples.

Gene	Group	*N* ^a^	*C*_*T*_^b^	*p* ^c^	*p* ^d^	AUC^e^
TTC3	Normal	21	4.77 ± 0.56	—		—
	Trisomy 18	6	4.81 ± 1.49	0.536	—	0.66 (0.36–0.96), *p* = 0.211
	**Trisomy 21**	**13**	**3.39 ± 0.99**	**<0.001**	**0.008**	**0.89 (0.74**–**1.00)**, ***p*** < **0.001**

RPL-17	Normal	21	2.19 ± 0.80	—		—
	**Trisomy 18**	**9**	**0.53 ± 0.53**	**<0.001**	—	**0.94 (0.86**–**1.00)**, ***p*** < **0.001**
	Trisomy 21	13	1.77 ± 0.87	0.400	0.002	0.50 (0.32–0.69), *p* = 0.968

^a^ *N* equals the number of embryos per a group. For TTC3, 3 embryos with Trisomy 18 had *C*_*T*_ values greater than 30 and were excluded from the analysis. ^b^Values are mean threshold cycle (*C*_*T*_) ± stand deviation; ^c^*p* value for the comparison between Trisomy embryos and normal embryos using ANOVA with a post hoc Bonferroni test; ^d^*p* value for the comparison between Trisomy 18 embryos and Trisomy 21 embryos using ANOVA with a post hoc Bonferroni test. ^e^The area under a Receiver-Operator Characteristic Curve (AUC) between Trisomy groups and the normal group. Values are expressed as AUC (95% confidence interval), *p* value.
