# Supplementary material for: Whole Genome Amplification of Day 3 or Day 5 Human Embryos Biopsies Provides a Suitable DNA Template for PCR-Based Techniques for Genotyping, a Complement of Preimplantation Genetic Testing
Source: Biomed Res Int. 2017 Jun 22;2017:1209158. doi: 10.1155/2017/1209158 (PMC5498896; doi:10.1155/2017/1209158)
Supplement: Supplementary file 1 — Supplementary Figure 1 illustrates CGH results, as well as melting curve analysis and cycle threshold difference for representative examples of Trisomy 18 and Trisomy 21 embryos. [file 1209158.f1.pdf]

Schaeffer et.al., 2016 Supplementary figure

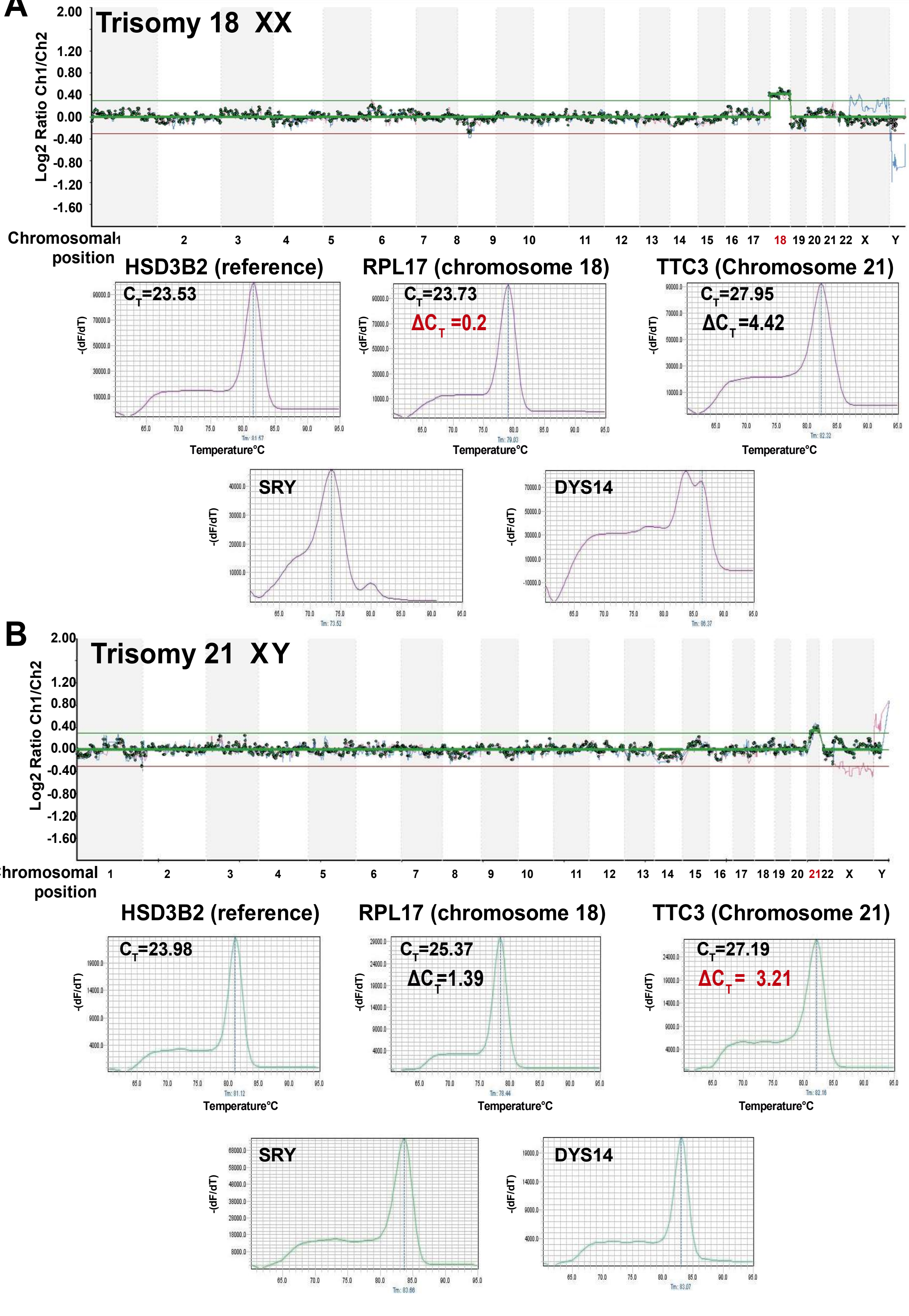

Supplementary figure

Detection of trisomies 18 and 21using CGH analysis and qPCR-based genotyping in blastocyst stage. CGH chromosome profiles and melting temperature peaks used for identification of (A) female (XX) embryo with trisomy 18 (B) male (XY) male embryo with trisomy 21. Delta C_T resulting from subtracting TTC3 and RPL17 from reference (HSD3B2) are exemplified.
